# Pollination of Cretaceous flowers

**DOI:** 10.1073/pnas.1916186116

**Published:** 2019-11-11

**Authors:** Tong Bao, Bo Wang, Jianguo Li, David Dilcher

**Affiliations:** ^a^State Key Laboratory of Palaeobiology and Stratigraphy, Nanjing Institute of Geology and Palaeontology, Chinese Academy of Sciences, 210008 Nanjing, China;; ^b^Center for Excellence in Life and Paleoenvironment, Chinese Academy of Sciences, 210008 Nanjing, China;; ^c^Institut für Geowissenschaften, Universität Bonn, 53115 Bonn, Germany;; ^d^Key Laboratory of Zoological Systematics and Evolution, Institute of Zoology, Chinese Academy of Sciences, 100101 Beijing, China;; ^e^Department of Geology and Atmospheric Science, Indiana University, Bloomington, IN 47405

**Keywords:** amber, insect, angiosperm, pollen, paleoecology

## Abstract

Insect pollination of flowering plants (angiosperms) is responsible for the majority of the world’s flowering plant diversity and is key to the Cretaceous radiation of angiosperms. Although both insects and angiosperms were common by the mid-Cretaceous, direct fossil evidence of insect pollination is lacking. Direct evidence of Cretaceous insect pollination is associated with insect-gymnosperm pollination. Here, we report a specialized beetle-angiosperm pollination mode from mid-Cretaceous Burmese amber (99 mega-annum [Ma]) in which a tumbling flower beetle (Mordellidae), *Angimordella burmitina* gen. et sp. nov., has many tricolpate pollen grains attached. *A. burmitina* exhibits several specialized body structures for flower-visiting behavior including its body shape and pollen-feeding mouthparts revealed by X-ray microcomputed tomography (micro-CT). The tricolpate pollen in the amber belongs to the eudicots that comprise the majority of extant angiosperm species. These pollen grains exhibit zoophilous pollination attributes including their ornamentation, size, and clumping characteristics. Tricolpate pollen grains attached to the beetle’s hairs are revealed by confocal laser scanning microscopy, which is a powerful tool for investigating pollen in amber. Our findings provide direct evidence of insect pollination of Cretaceous angiosperms, extending the range insect-angiosperm pollination association by at least 50 million years. Our results support the hypothesis that specialized insect pollination modes were present in eudicots 99 million years ago.

## Significance

Since Darwin, insect pollination was thought to be a key contributor to the Cretaceous radiation of angiosperms. Both insects and angiosperms were common during the mid-Cretaceous, but direct evidence for a Cretaceous insect-angiosperm pollination mode was until now absent. Here, we report a specialized beetle-angiosperm pollination mode preserved in Burmese amber where a tumbling flower beetle is carrying tricolpate pollen grains that belongs to the eudicots that comprise the majority of extant angiosperm species. Our study provides direct evidence of insect pollination of Cretaceous flowers, which is further supported by the flower-visiting body shape, specialized pollen-feeding mouthparts, and zoophilous pollen grains. These findings demonstrate that insect pollination of flowering plants was well established 99 million years ago.

Angiosperms, flowering plants, are the most diverse group of land plants (1). The earliest unequivocal pollen and macrofossils of angiosperms are generally thought to date from the early Hauterivian (∼130 Ma) and early Aptian (∼125 Ma), respectively, (2, 3) despite claims based on other fossils and molecular analyses (1, 4, 5). The apparently rapid and tremendous evolutionary diversification of angiosperms during the Cretaceous was the great “abominable mystery” mentioned by Darwin and continues to be an active and sometimes a controversial area of research (6–8). Insect pollination (entomophily) is generally considered to be a key contributor to the Cretaceous radiation of angiosperms (9–12). It is generally thought to be the dominant pollination mode of angiosperms during the early mid-Cretaceous with specialization increasing during the angiosperm radiation, supported by basal flower morphology, palynological data, and phylogenetic inferences (8, 13–15). Some Cretaceous insects are palynivores of angiosperms based on their pollen- or nectar-feeding mouthparts (16, 17), gut contents (18), or coprolites (19). However, a palynivore is not equivalent to a pollinator. Only direct evidence (pollen-carrying behavior and pollen-feeding mouthparts) can provide unambiguous demonstration of ancient insect pollination. Until now, direct evidence of Cretaceous insect pollination supports insect-gymnosperm pollination, such as that involving thrips (20), true flies (21), beetles (22, 23), and scorpionflies (17). Although both insects and angiosperms were common during the mid-Cretaceous, direct evidence for Cretaceous insect-angiosperm pollination mode has been absent.

The Coleoptera (beetles) constitute almost 1-4th of all animal species on Earth (24) and are among the most prominent pollinators of angiosperms (25, 26). More than 77,000 beetle species are estimated to visit flowers (27). Among these flower-visiting beetles, Mordellidae (tumbling flower beetles) is one of the most species-rich families, and adults are easily recognized by their humpbacked body, deflexed head, pointed abdomen, and stout hind legs (28, 29). The majority of extant adult mordellids feed on angiosperm pollen (28, 29). Cretaceous mordellids have been hypothesized to be angiosperm pollinators, but direct evidence was lacking (30).

We report a species of Mordellidae from mid-Cretaceous Burmese amber (see *Systematic Descriptions and Notes*). We used optical microscopy, confocal laser scanning microscopy (CLSM), and X-ray microcomputed tomography (micro-CT) to reveal the morphology of the pollen and beetle mouthparts. Multiple lines of evidence, including pollen-feeding mouthparts, pollen-carrying hairs on the body, and zoophilous pollination attributes of these tricolpate pollen, strongly support a specialized beetle-angiosperm pollination mode. This is the earliest direct evidence of insect pollination of angiosperms.

## Discussion

**Fig. 1.**
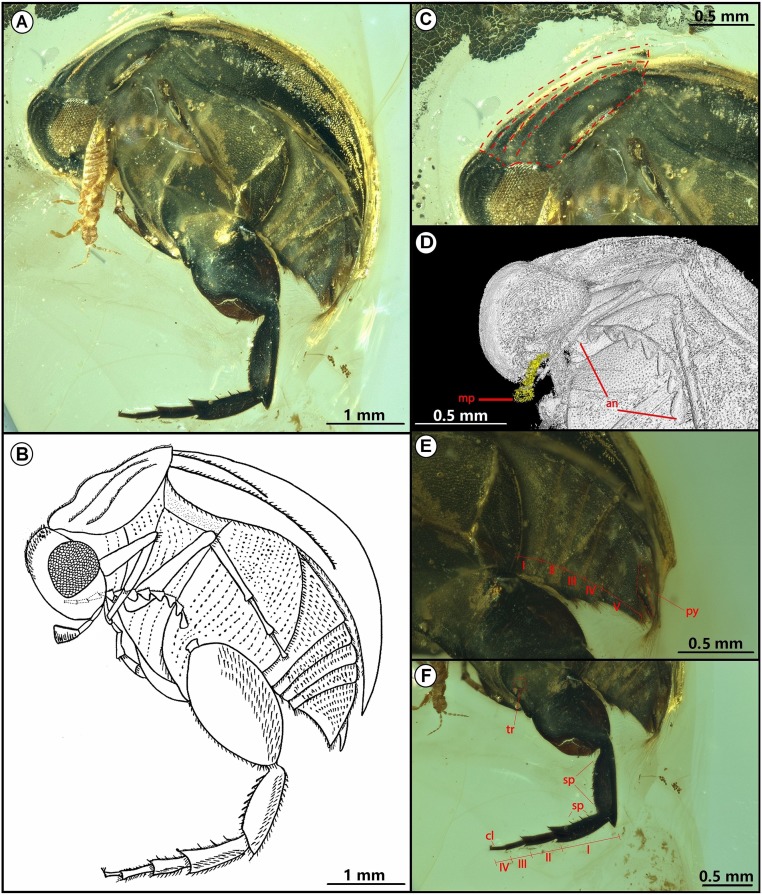
Cretaceous tumbling flower beetle *A. burmitina*. (*A*) Habitus. (*B*) Drawing. (*C*) Prothorax and pronotum highlighted by red dashed lines. (*D*) Microtomographic reconstruction of the head. Maxillary palpi highlighted in yellow. (*E*) Abdomen, I−IV represent first to fifth abdominal ventrites. (*F*) Hind leg, I−IV represent first to fourth metatarsomeres. an, antennae; cl, claw; mp, maxillary palp; py, pygidium; sp, spines on metatibiae and metatarsi; tr, trochanter.

**Fig. 2.**
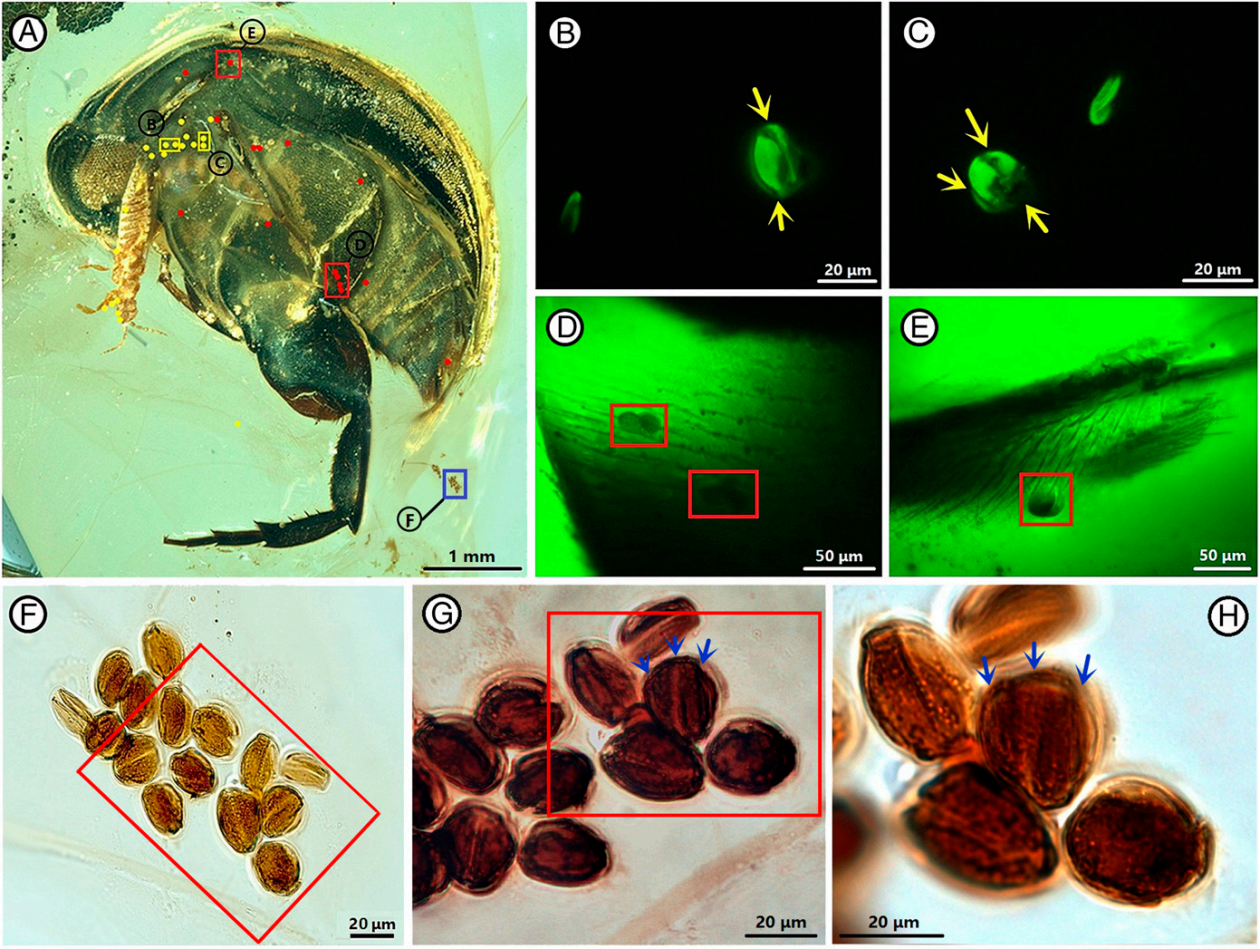
*A. burmitina* and tricolpate pollen grains. (*A*) Habitus. Pollen grains attached to the body are indicated by red dots, unattached are indicated by yellow dots, clumped pollen are indicated by blue squares. (*B*−*H*) Locations are highlighted in *A*. (*B* and *C*) Pollen grains near the body. Yellow arrows point to colpi. (*D* and *E*) Pollen grains on the body. (*F*−*H*) Clumped pollen grains. (*G* and *H*) Locations are highlighted in *F* and *G*, respectively. Blue arrows point to colpi.

*Angimordella burmitina* exhibit a series of specialized body structures related to its flower-visiting behavior, similar to its modern counterparts, which feed on various angiosperm pollen (28, 29). It has the *Mordella*-type apical maxillary palpomere, which is enlarged and securiform (31). This maxillary palpomere is blocked by a thrip, but the palpomere shape is revealed by micro-CT (Fig. 1; see Movie S1 for detailed account). This specialized modification of the maxillary palpomere has been known to aid collecting and most likely transporting pollen grains (30, 32). *A. burmitina* has a curved and laterally compressed body with a strongly declined head, allowing for flexibility when feeding inside the flower (31). Its hind legs are well developed, with enlarged metacoxa and metafemora and spiny metatibiae and metatarsi, which make it easier to move on the corolla and from one flower to another (33). Moreover, *A. burmitina* has fine hairs; the spacing and height of these hairs influence the ability of the hairs to carry pollen grains (34) (Fig. 2 *D* and *E*). Accordingly, the hairs on the beetle’s thorax and abdominal sternites are distinctly longer than 30 µm, and the spacing between the hairs is consistent with the width of the coexistent pollen grains (∼20 µm) and is well-adapted for holding and transporting pollen grains (34).

It is important to note that *A. burmitina* is covered by abundant tricolpate pollen grains that are mainly distributed on the thorax and abdomen (Fig. 2*A*). Tricolpate pollen is both the defining and most important character of the eudicots. The eudicots comprise ∼75% of extant angiosperm species (35). The earliest fossil record of tricolpate pollen is ∼125 million years old, slightly older than the earliest eudicot macrofossil (36). By 99 million years ago (Burmese amber age), tricolpate pollen had become widespread worldwide (5, 37–39) and eudicot macrofossils are reported from Burmese amber (e.g., refs. 40 and 41). Many Cretaceous plants with tricolpate pollen are animal-pollinated and characterized by their ornamentation, size (10−300 µm), and clumping characteristics (15). Small angiosperm pollen grains in amber, especially those buried under insect body hairs, are often not visible under optical microscopy and, thus, could be easily overlooked. In this study, the pollen grains between body hairs were detected by confocal laser scanning microscopy (CLSM), which takes advantage of pollen fluorescence, which contrasts with the surrounding dark insect cuticle (41) (Fig. 2 *D* and *E*). The tricolpate pollen grains found in Burmese amber exhibit remarkable zoophilous pollination features including their reticulate surface (Fig. 2*H*) and presence of pollen clumping (Fig. 2*F*), thus providing more evidence to support beetle-mediated pollination. Interestingly, only one type of pollen was found on this beetle. This could reflect that there were not very many different types of flowers during the mid-Cretaceous or that the insect visited only one type of flower before it was trapped in the amber.

**Fig. 3.**
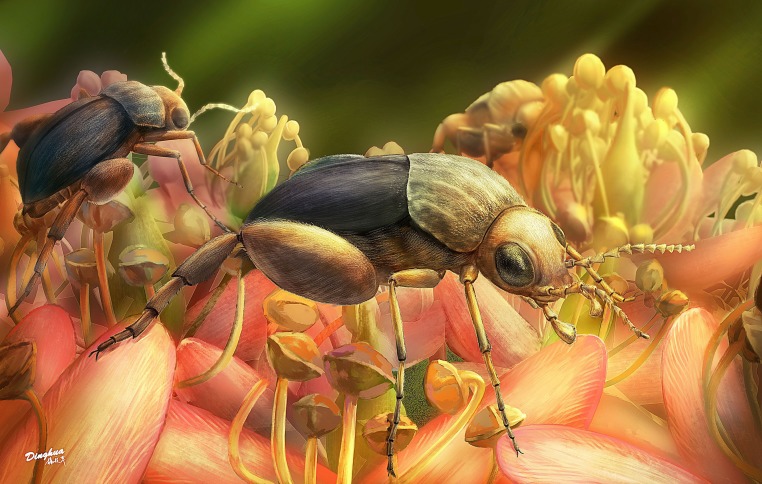
Ecological reconstruction of *A. burmitina*. These tumbling flower beetles are feeding on eudicot flowers. The color and morphology of flowers are artistic only.

Mordellidae, comprising ∼1,500 extant species worldwide, are among the most basal group of Tenebrionoidea based on morphological analysis and molecular data (24, 42, 43). Although mordellid-like beetles are reported from the Middle-Late Jurassic of China and Kazakhstan, the earliest true mordellids (extant subfamily) are known from the mid-Cretaceous Spanish and Burmese amber (44). *A. burmitina* is among the earliest true mordellids and indicates that mordellid-angiosperm pollination mutualisms have been present since at least 99 million years ago (Fig. 3). These mutualisms may be an important driver for the radiation of true mordellids.

This study provides direct evidence of Cretaceous insect pollination of angiosperms, which is strongly supported by the flower-visiting body shape, specialized pollen-feeding mouthparts, and zoophilous pollen grains attached to the body. The prior earliest direct evidence of insect pollination of angiosperms was reported from several pollen-collection bees from the middle Eocene of Eckfeld and Messel (48 and 45 Ma, respectively) in Germany (45). Our finding thereby extends the known geological range of direct evidence of insect pollination of angiosperm by at least 50 million years.

## Systematic Descriptions and Notes

Family Mordellidae Latreille, 1802.

Subfamily Mordellinae Latreille, 1802.

*Angimordella burmitina* gen. et sp. nov.

### Etymology.

The generic name is derived from the Latin prefix “angi” (referring to angiosperm) and the genus *Mordella* Linnaeus. The specific name is derived from Latin “Burmitina,” referring to the mineralogical name of Burmese amber.

### Holotype.

NIGP171315 (Fig. 1), a complete beetle with left side visible but its right side covered by abundant microbubbles. A thrip is near the maxillary palpi of the beetle on the left side.

### Horizon and Locality.

Mid-Cretaceous (∼99 Ma); Burmese amber, from the Hukawng Valley, Kachin State, Myanmar.

### Diagnosis.

Body small, with pronotum and elytra with wrinkles or ridges dorsally; antennae serrate; mesotibiae and metatibiae without any kind of ridge including subapical one; pygidium not well developed, shorter than 1/2 of last abdominal sternite.

### Description.

Body strongly convex, wedge-shaped, widest near base of prothorax, slightly narrowed anteriorly and posteriorly (Fig. 1 *A* and *B*). Body length 4.25 mm; ratio of body length to greatest body width 3:2. Head length 0.51 mm, large, transverse, strongly declined, with mouthparts directly posteriorly; compound eyes finely faceted and glabrous. Occipital region wide, surface with wrinkles and hairs, matching perfectly with anterior edge of pronotum. Antennae comparatively short, with 7 visible antennomeres, obviously serrate (Fig. 1*D*), covered with hairs. Maxillary palp length 0.38 mm; apical maxillary palpomere length 0.18 mm, securiform, strongly enlarged.

Pronotum length 1.11 mm, slightly narrowed anteriorly, widest posteriorly, as wide as elytra at base, lateral margin slightly curved; pronotum disk with ridges and covered by short and dense recumbent hairs (Fig. 1*C*). Elytra length 2.46 mm, ∼2.5 times as long as pronotum, covering all abdominal segments, with slight surface relief, gradually curving up to apex proximity; integuments covered with fine hairs. Forelegs simple, tibiae slender. Mesotibiae and mesotarsi simple, long and slender. Metaepistena long, rectangular. Metacoxae greatly enlarged with rounded posterior margin, extending laterally to meet elytra, widely rounded at posterior margin; trochanter oblique. Metafemora length 0.80 mm, laterally compressed and greatly expanded, more than 4 times wider than mesofemora. Metatibiae length 0.74 mm, blade shaped, about same length as metafemora with obliquely truncated apexes; metatibiae without any kind of ridge including subapical one; apical margin of hind tibiae bearing comb-like setae; ventral side of metatibiae and metatarsomeres with fine spine-like setae; apical spurs on metatibiae absent. Metatarsi laterally compressed, comparatively sturdy, with comb-like setae on apical margin and apical spurs on posterior margin, spiny on inner margins (Fig. 1*F*); length of four metatarsomeres 0.50 mm, 0.30 mm, 0.18 mm, 0.16 mm, ratio 5:3:2:2. Claws small and bicleft. Abdomen distinctly narrowed posteriorly, with 5 free ventrites. Ventrites 1−5 length 0.16 mm, 0.16 mm, 0.15 mm, 0.12 mm, 0.26 mm, ratio 1:1:1:1:2 (Fig. 1*E*). Hairs present on abdomen, slightly elongated between sternites. Pygidium very short, 0.18 mm long.

### Remarks.

*A. burmitina* can be attributed to the subfamily Mordellinae by the following characters: wedge-shaped body, enlarged last segment of maxillary palpi; metacoxae greatly enlarged forming a rounded plate; metafemora expanded and well developed; and pygidium very short. It resembles *Primaevomordellida burmitina* Bao et al. 2019 from Burmese amber (46) in the absence of ridge on metatibiae and metatarsi but differs from the latter in having a short pointed pygidium. It is also similar to *Mediumiuga sinespinis* Peris & Ruzzier, 2013 from late Albian Spanish amber (47) in having a very short pygidium and ventrally spiny metatibiae and metatarsi but differs from the latter in the absence of ridge on metatibiae and metatarsi.

### Pollen Descriptions.

There are at least 62 pollen grains (from only the visible left side of the beetle) in the amber in total, of which 24 pollen grains aggregate into two small clusters near the abdominal end of the mordellid (Fig. 2*A*). Pollen grains in the amber are retitricolpate and highly uniform in morphology (Fig. 2 *B*, *C*, and *H*). The shape of the grain is approximately oblate spheroidal, 25.56 μm (30.95−22.08 μm) × 16.49 μm (20.68−13.93 μm) in equatorial view (based on measurement of the 27 best preserved pollen grains; *SI Appendix*, Table S1). The colpi are long, wide, and deep and extend to the pole. The exine is moderately thick, ∼1 μm. The lumina are small, evenly spaced, and ∼0.5 μm in diameter. The pollen clump shape is irregular, and the pollen grains are well preserved (Fig. 2 *F* and *G*), indicating that they are natural floral remains rather than coprolites (48). These pollen grains can be confidently attributed to the eudicot monophyletic group (true dicotyledons), members of which are distinguished from all other angiosperms by their tricolpate pollen structure (49, 50). We did not assign the pollen to a taxon given the nature of this microscopic method conducted within amber.

## Materials and Methods

### Materials.

The amber piece came from an amber mine near Noije Bum Village, Danai Town in northern Myanmar. The U-Pb dating of zircons from the volcanoclastic matrix of the amber gave a maximum age of 98.8 ± 0.6 million years (51), which is also supported by the ammonite trapped in the amber (52). The specimen (NIGP171315) is deposited in the Nanjing Institute of Geology and Paleontology (NIGPAS), Chinese Academy of Sciences.

### Optical Photomicrography.

Photographs were taken using a Zeiss AXIO Zoom V16 microscope system at the State Key Laboratory of Paleobiology and Stratigraphy, NIGPAS. Incident and transmitted light were used simultaneously in most instances. Each image was digitally stacked with 40−50 individual focal planes, produced with the software Helicon Focus 6 (https://www.heliconsoft.com/) for better illustration of the 3-dimensional (3D) structures.

### Confocal Laser Scanning Microscopy.

Photomicrographs with green background were taken using a CLSM Zeiss LSM710 system with laser wavelength 488 nm (Laser module LGK 7812 ML5) at the State Key Laboratory of Paleobiology and Stratigraphy, NIGPAS. Based on the diameter and thickness of amber specimen, 2 objectives (“Plan-Neofluar” 20×/0.50 M27 and “Plan-Apochromat” 63×/1.40 Oil DIC M27) were applied. AxioVision 4.0 modules with the software AxioVision Rel. 4.8.2 were used to produce high-resolution images.

### X-ray Microcomputed Tomography.

To 3-dimensionally reconstruct the beetle, we scanned the fossil at the micro-CT laboratory of NIGPAS, using a 3D X-ray microscope (3D-XRM), Zeiss Xradia 520 versa. Unlike conventional micro-CT, which relies on maximum geometric magnification and a flat panel detector to achieve high resolution, 3D-XRM uses charge-coupled device (CCD)-based objectives to achieve higher spatial resolution. Based on the size of the fossil specimen, a CCD and 4× objective was used, providing isotropic voxel sizes of 3.43 μm with the help of geometric magnification. During the scan, the acceleration voltage for the X-ray source was 50 kV (power 4W), and a thin filter (LE3) was used to avoid beam hardening artifacts. To improve signal-to-noise ratio, 3,000 projections over 360° were collected, and the exposure time for each projection was 5 s. Volume data processing was performed using software VGStudio Max (version 3.0, Volume Graphics, Heidelberg, Germany).

### Nomenclatural Acts.

This published work and the nomenclatural acts it contains have been registered in ZooBank, the proposed online registration system for the International Code of Zoological Nomenclature. The ZooBank LSIDs (Life Science Identifiers) can be resolved and the associated information viewed through any standard web browser by appending the LSID to the prefix http://zoobank.org/. The LSIDs for this publication are as follows: urn:lsid:zoobank.org:pub: 2CE49289-946F-4194–904B-188A29976905; urn:lsid:zoobank.org:act:D31C312B-218A-47EC-A427-53ED39FE1926; urn:lsid:zoobank.org:act:46B77E87-5047–49B4-B29A-448D0B41CA9D.

### Data Availability.

The data supporting the findings of this study have been deposited in the Figshare database (53) and can be obtained upon request from the corresponding authors.

## Supplementary Material

Supplementary File

Supplementary File
