# Supplementary material for: Pollination of Cretaceous flowers
Source: Proc Natl Acad Sci U S A. 2019 Nov 11;116(49):24707–11. doi: 10.1073/pnas.1916186116 (PMC6900596; doi:10.1073/pnas.1916186116)
Supplement: Supplementary File [file pnas.1916186116.sapp.pdf]

Table S1. Length and width measurements of pollen grains.

www.pnas.org/cgi/doi/10.1073/pnas.1916186116

Table S1. Length and width measurements of pollen grains.

Length (μm)	Width (μm)
22.50	14.31
25.83	17.92
27.54	19.29
23.80	20.01
23.06	16.36
23.90	16.46
24.44	16.17
22.08	20.68
23.60	16.86
22.75	15.91
27.05	17.06
30.95	15.90
22.55	18.17
27.13	18.57
24.98	17.39
28.40	15.38
26.95	15.77
25.11	14.53
28.89	18.31
27.12	14.42
29.83	19.39
23.98	14.05
25.79	14.33
22.24	15.01
25.71	14.40
23.41	13.93
30.58	14.80

Note: Pollen length is the maximum value between two polar points, and pollen width is the maximum value of the equatorial zone.

Video S1 Legend: Micro-tomographic reconstruction of Cretaceous tumbling flower beetle *Angimordella burmitina*.
